# Supplementary material for: Incidence of depression and anxiety in children and adolescents following COVID-19 infection
Source: PLoS One. 2025 Sep 8;20(9):e0331984. doi: 10.1371/journal.pone.0331984 (PMC12416683; doi:10.1371/journal.pone.0331984)
Supplement: S1 Appendix — (DOCX) [file pone.0331984.s001.docx]

Appendix A. Standardized mean differences before and after entropy balancing

Before entropy balancing

|  | Subjects with COVID-19 infection | | | Subjects with no COVID-19 infection | | |  |
| --- | --- | --- | --- | --- | --- | --- | --- |
|  | mean | variance | skewness | mean | variance | skewness | standardized mean difference |
| Age category | 0.559 | 0.247 | -0.239 | 0.466 | 0.249 | 0.138 | 0.189 |
| Female | 0.483 | 0.250 | 0.069 | 0.473 | 0.249 | 0.108 | 0.019 |
| Hispanic | 0.082 | 0.075 | 3.048 | 0.097 | 0.088 | 2.726 | -0.054 |
| Non-Hispanic Black | 0.006 | 0.006 | 12.720 | 0.011 | 0.011 | 9.349 | -0.064 |
| Non-Hispanic others | 0.017 | 0.017 | 7.374 | 0.018 | 0.018 | 7.269 | -0.004 |
| Unknown | 0.755 | 0.185 | -1.183 | 0.729 | 0.198 | -1.029 | 0.060 |
| Medicaid | 0.262 | 0.193 | 1.083 | 0.330 | 0.221 | 0.722 | -0.155 |
| Sleep disorder | 0.025 | 0.024 | 6.106 | 0.017 | 0.017 | 7.429 | 0.049 |
| ADHD | 0.051 | 0.048 | 4.103 | 0.044 | 0.042 | 4.446 | 0.030 |
| Pain | 0.212 | 0.167 | 1.409 | 0.148 | 0.126 | 1.985 | 0.157 |
| Blindness | 0.025 | 0.024 | 6.094 | 0.024 | 0.024 | 6.161 | 0.003 |
| Cancer | 0.074 | 0.069 | 3.253 | 0.057 | 0.054 | 3.822 | 0.065 |
| Asthma | 0.074 | 0.069 | 3.251 | 0.046 | 0.044 | 4.329 | 0.107 |
| Headache | 0.045 | 0.043 | 4.403 | 0.031 | 0.030 | 5.415 | 0.067 |
| Severe allergy | 0.103 | 0.092 | 2.610 | 0.080 | 0.073 | 3.109 | 0.078 |
| Hearing loss | 0.014 | 0.014 | 8.222 | 0.009 | 0.009 | 10.130 | 0.040 |
| Neurocognitive disorders | 0.080 | 0.074 | 3.088 | 0.072 | 0.067 | 3.322 | 0.032 |
| Obesity | 0.068 | 0.063 | 3.446 | 0.055 | 0.052 | 3.899 | 0.050 |

Appendix A. Standardized mean differences before and after entropy balancing (continued)

After entropy balancing

|  | Subjects with COVID-19 infection | | | Subjects with no COVID-19 infection | | |  |
| --- | --- | --- | --- | --- | --- | --- | --- |
|  | mean | variance | skewness | mean | variance | skewness | standardized mean difference |
| Age category | 0.559 | 0.247 | -0.239 | 0.559 | 0.247 | -0.238 | 0.001 |
| Female | 0.483 | 0.250 | 0.069 | 0.483 | 0.250 | 0.069 | 0.000 |
| Hispanic | 0.082 | 0.075 | 3.050 | 0.082 | 0.075 | 3.048 | 0.000 |
| Non-Hispanic Black | 0.006 | 0.006 | 12.720 | 0.006 | 0.006 | 12.670 | -0.001 |
| Non-Hispanic others | 0.017 | 0.017 | 7.380 | 0.017 | 0.017 | 7.380 | 0.000 |
| Unknown | 0.755 | 0.185 | -1.183 | 0.754 | 0.185 | -1.185 | 0.000 |
| Medicaid | 0.262 | 0.193 | 1.083 | 0.262 | 0.193 | 1.082 | -0.001 |
| Sleep disorder | 0.025 | 0.024 | 6.110 | 0.025 | 0.024 | 6.111 | 0.000 |
| ADHD | 0.051 | 0.048 | 4.103 | 0.051 | 0.048 | 4.106 | 0.000 |
| Pain | 0.212 | 0.167 | 1.409 | 0.212 | 0.167 | 1.410 | 0.000 |
| Blindness | 0.025 | 0.024 | 6.093 | 0.025 | 0.024 | 6.093 | 0.000 |
| Cancer | 0.074 | 0.069 | 3.253 | 0.074 | 0.069 | 3.254 | 0.000 |
| Asthma | 0.074 | 0.069 | 3.251 | 0.074 | 0.069 | 3.252 | 0.000 |
| Headache | 0.045 | 0.043 | 4.403 | 0.045 | 0.043 | 4.404 | 0.000 |
| Severe allergy | 0.103 | 0.092 | 2.610 | 0.103 | 0.092 | 2.611 | 0.000 |
| Hearing loss | 0.014 | 0.014 | 8.223 | 0.014 | 0.014 | 8.224 | 0.000 |
| Neurocognitive disorders | 0.080 | 0.074 | 3.088 | 0.080 | 0.074 | 3.088 | 0.000 |
| Obesity | 0.068 | 0.063 | 3.446 | 0.068 | 0.063 | 3.446 | 0.000 |
