# Supplementary material for: Incidence of depression and anxiety in children and adolescents following COVID-19 infection
Source: PLoS One. 2025 Sep 8;20(9):e0331984. doi: 10.1371/journal.pone.0331984 (PMC12416683; doi:10.1371/journal.pone.0331984)
Supplement: Attachment — Submitted filename: Completed PONE-D-25-32255_reviewer.pdf [file pone.0331984.s002.pdf]

PLOS ONE

Incidence of depression and anxiety in children and adolescents following COVID-19 infection

--Manuscript Draft--

**Manuscript Number:**	PONE-D-25-32255
**Article Type:**	Research Article
**Full Title:**	Incidence of depression and anxiety in children and adolescents following COVID-19 infection
**Short Title:**	COVID and depression
**Corresponding Author:**	Jaewhan Kim, Ph.D.<br>University of Utah Health<br>Salt Lake City, Utah UNITED STATES OF AMERICA
**Keywords:**	COVID-19; depression; Anxiety; Children; Adolescents
**Abstract:**	While the COVID-19 pandemic has significantly impacted mental health, limited information exists on the relationship between COVID-19 infection and mental health disorders in children and adolescents. This study explored the association between COVID-19 infection, infection severity, and the risk of depression and anxiety among school-aged youth. Data from the 2019–2021 Utah All Payers Claims Database (APCD) were used to identify children and adolescents (aged 6–15 years in 2019) covered by private insurance or Medicaid. Depression and anxiety diagnoses in 2021 were identified using diagnosis codes. Entropy balancing was applied to balance baseline differences between youth with and without a COVID-19 infection in 2020. Weighted logistic regression was conducted to identify factors associated with incidence of depression and anxiety. A total of 154,579 subjects were included, with an average age of 10.8 years (SD: 2.8); 48% were female. Youth with a COVID-19 infection had a 49% higher likelihood of having new depression or anxiety diagnoses in 2021 (OR = 1.49, p < 0.01). Compared to youth without COVID-19 infection, those with moderate symptoms had a 40% higher likelihood (OR = 1.40, p < 0.01), and those with severe symptoms had a 60% higher likelihood (OR = 1.60, p < 0.01) of developing depression or anxiety. This population-level study found that COVID-19 infection, especially severe cases, is associated with an increased risk of depression and anxiety in children and adolescents. These findings underscore the importance of targeted mental health interventions for youth impacted by the infection.
**Order of Authors:**	Jaewhan Kim, Ph.D.
	Carson R Ewing, BS
	Ashlee Larson, BS
	Chathuri Illapperuma-Wood, Ph.D.
	Aaron Fischer, Ph.D.
	Emeka Elvis Duru, BPharm
	Youngwoo Kim, BS
	Fernando Wilson, Ph.D.
**Additional Information:**	
**Question**	**Response**
**Financial Disclosure**<br><br>Enter a financial disclosure statement that describes the sources of funding for the work included in this submission. Review the submission guidelines for detailed	The author(s) received no specific funding for this work.

Powered by Editorial Manager® and ProduXion Manager® from Aries Systems Corporation

requirements. View published research articles from *PLOS ONE* for specific examples.<br><br>This statement is required for submission and **will appear in the published article** if the submission is accepted. Please make sure it is accurate.<br><br>**Funded studies**<br>Enter a statement with the following details:<br>• Initials of the authors who received each award<br>• Grant numbers awarded to each author<br>• The full name of each funder<br>• URL of each funder website<br>• Did the sponsors or funders play any role in the study design, data collection and analysis, decision to publish, or preparation of the manuscript?<br><br>Did you receive funding for this work?	
**Competing Interests**<br><br>Use the instructions below to enter a competing interest statement for this submission. On behalf of all authors, disclose any competing interests that could be perceived to bias this work—acknowledging all financial support and any other relevant financial or non-financial competing interests.<br><br>This statement is required for submission and **will appear in the published article** if the submission is accepted. Please make sure it is accurate and that any funding sources listed in your Funding Information later in the submission form are also declared in your Financial Disclosure statement.<br><br>View published research articles from *PLOS ONE* for specific examples.	The authors have declared that no competing interests exist.

**NO authors have competing interests**<br><br>Enter: *The authors have declared that no competing interests exist.*<br><br>**Authors with competing interests**<br><br>Enter competing interest details beginning with this statement:<br><br>*I have read the journal's policy and the authors of this manuscript have the following competing interests: [insert competing interests here]*<br><br>* typeset	
**Ethics Statement**<br><br>Enter an ethics statement for this submission. This statement is required if the study involved:<br><br>• Human participants<br>• Human specimens or tissue<br>• Vertebrate animals or cephalopods<br>• Vertebrate embryos or tissues<br>• Field research<br><br>Write "N/A" if the submission does not require an ethics statement.<br><br>General guidance is provided below. Consult the submission guidelines for detailed instructions. **Make sure that all information entered here is included in the Methods section of the manuscript.**	The study was conducted in accordance with the Declaration of Helsinki, and approved by the Institutional Review Board (or Ethics Committee) of University of Utah (protocol code IRB_00151091 and November 09, 2023).

**Format for specific study types**<br><br>**Human Subject Research (involving human participants and/or tissue)**<br>• Give the name of the institutional review board or ethics committee that approved the study<br>• Include the approval number and/or a statement indicating approval of this research<br>• Indicate the form of consent obtained (written/oral) or the reason that consent was not obtained (e.g. the data were analyzed anonymously)<br><br>**Animal Research (involving vertebrate animals, embryos or tissues)**<br>• Provide the name of the Institutional Animal Care and Use Committee (IACUC) or other relevant ethics board that reviewed the study protocol, and indicate whether they approved this research or granted a formal waiver of ethical approval<br>• Include an approval number if one was obtained<br>• If the study involved *non-human primates*, add *additional details* about animal welfare and steps taken to ameliorate suffering<br>• If anesthesia, euthanasia, or any kind of animal sacrifice is part of the study, include briefly which substances and/or methods were applied<br><br>**Field Research**<br><br>Include the following details if this study involves the collection of plant, animal, or other materials from a natural setting:<br>• Field permit number<br>• Name of the institution or relevant body that granted permission	
**Data Availability**<br><br>Authors are required to make all data underlying the findings described fully available, without restriction, and from the time of publication. PLOS allows rare exceptions to address legal and ethical concerns. See the PLOS Data Policy and FAQ for detailed information.	No - some restrictions will apply

A Data Availability Statement describing where the data can be found is required at submission. Your answers to this question constitute the Data Availability Statement and **will be published in the article**, if accepted.<br><br>**Important:** Stating 'data available on request from the author' is not sufficient. If your data are only available upon request, select 'No' for the first question and explain your exceptional situation in the text box.<br><br>Do the authors confirm that all data underlying the findings described in their manuscript are fully available without restriction?	
**Describe where the data may be found in full sentences. If you are copying our sample text, replace any instances of XXX with the appropriate details.**<br><br>• If the data are **held or will be held in a public repository**, include URLs, accession numbers or DOIs. If this information will only be available after acceptance, indicate this by ticking the box below. For example: *All XXX files are available from the XXX database (accession number(s) XXX, XXX.).*<br>• If the data are all contained **within the manuscript and/or Supporting Information files**, enter the following: *All relevant data are within the manuscript and its Supporting Information files.*<br>• If neither of these applies but you are able to provide **details of access elsewhere**, with or without limitations, please do so. For example:<br><br>*Data cannot be shared publicly because of [XXX]. Data are available from the XXX Institutional Data Access / Ethics Committee (contact via XXX) for researchers who meet the criteria for access to confidential data.*<br><br>*The data underlying the results presented in the study are available from (include the name of the third party*	Data cannot be shared publicly because of the regulations of Utah Department of Health and Human Services, and the Utah Resource for Genetic and Epidemiologic Research (RGE). Data are available from the University of Utah Institutional Data Access / Ethics Committee (contact via RGE) for researchers who meet the criteria for access to confidential data.

and contact information or URL).<br>• This text is appropriate if the data are owned by a third party and authors do not have permission to share the data.<br><br>* typeset	
Additional data availability information:	

manuscript Click here to access/download;Manuscript;manuscript.docx

Incidence of depression and anxiety in children and adolescents following COVID-19 infection

Short title: COVID and depression

Jaewhan Kim[1*], Carson R. Ewing[2], Ashlee Larson[2], Chathuri Illapperuma-Wood[2], Aaron Fischer[2], Emeka Elvis Duru[3], Youngwoo Kim[2], Fernando Wilson[4]

[1] Department of Physical Therapy, University of Utah, Salt Lake City, Utah, United States of America
[2] Department of Educational Psychology, University of Utah, Salt Lake City, Utah, United States of America
[3] Department of Pharmacotherapy, University of Utah, Salt Lake City, Utah, United States of America
[4] Department of Population Health Sciences, University of Utah, Salt Lake City, Utah, United States of America

[*] Corresponding author

Email: Jaewhan.kim@utah.edu (JK)

Abstract

While the COVID-19 pandemic has significantly impacted mental health, limited information exists on the relationship between COVID-19 infection and mental health disorders in children and adolescents. This study explored the association between COVID-19 infection, infection severity, and the risk of depression and anxiety among school-aged youth. Data from the 2019–2021 Utah All Payers Claims Database (APCD) were used to identify children and adolescents (aged 6–15 years in 2019) covered by private insurance or Medicaid. Depression and anxiety diagnoses in 2021 were identified using diagnosis codes. Entropy balancing was applied to balance baseline differences between youth with and without a COVID-19 infection in 2020. Weighted logistic regression was conducted to identify factors associated with incidence of depression and anxiety. A total of 154,579 subjects were included, with an average age of 10.8 years (SD: 2.8); 48% were female. Youth with a COVID-19 infection had a 49% higher likelihood of having new depression or anxiety diagnoses in 2021 (OR = 1.49, $p < 0.01$). Compared to youth without COVID-19 infection, those with moderate symptoms had a 40% higher likelihood (OR = 1.40, $p < 0.01$), and those with severe symptoms had a 60% higher likelihood (OR = 1.60, $p < 0.01$) of developing depression or anxiety. This population-level study found that COVID-19 infection, especially severe cases, is associated with an increased risk of depression and anxiety in children and adolescents. These findings underscore the importance of targeted mental health interventions for youth impacted by the infection.

Keywords: COVID-19, depression, anxiety, children, adolescents

Introduction

The prevalence of mental health disorders among youth has surged during the COVID-19 pandemic, with significant increases in depression and anxiety. The proportion of youth experiencing a major depressive episode rose from 9.1% in 2019 to 18.33% in 2023, as reported by Mental Health America [1, 2]. The rise in mental illness among children (aged 6–11) and adolescents (aged 12–17) during this period is likely a result of several interconnected factors. Key contributors include the pandemic's direct impact on daily life, such as school closures, isolation from peers, and disruptions to family routines. These challenges have likely had long-term consequences for the mental health and well-being of young people [3, 4]. Moreover, COVID-19 infection itself may play a significant role in the development of mental health disorders among children and adolescents. Although the relationship between COVID-19 infection and mental health outcomes in youth remains inconclusive, prior research has shown varying results. Some studies suggest that contracting COVID-19 may elevate the risk of mental health disorders, including anxiety and depression, among youth [5, 6]. Research has shown that children and adolescents who contracted COVID-19 may have been more likely to experience heightened mental health challenges [7]. For instance, one study found that children with pre-existing mental health conditions experienced exacerbated symptoms following COVID-19 infection [8].

However, other studies have reported no significant relationship between COVID-19 infection and mental health outcomes in children [9, 10], with one study suggesting the need for a holistic approach to adolescent mental health, asserting that there is no clear evidence linking COVID-19 infection to an increased risk of mental health problems [10].

Despite the potential impact of COVID-19 on youth mental health, a notable gap exists in the literature exploring the relationship between COVID-19 infection and subsequent mental health

outcomes in children and adolescents. Previous studies have often involved small sample sizes, limiting the generalizability of their findings, and many have overlooked the severity of COVID-19 infection as a potential factor influencing the development of mental health disorders in this population [7].

Children and adolescents are at a critical stage of emotional, social, and cognitive development, and disruptions during this period can have profound and long-lasting effects on academic performance, mental health, and overall well-being [11]. Understanding the role that COVID-19 infection might play in exacerbating mental health issues during this developmental phase is crucial for developing targeted interventions and support mechanisms for affected youth.

Given these gaps, this study aims to examine the potential causal relationship between COVID-19 infection, its severity, and the associated risks of depression and anxiety among school-aged children. By utilizing a state-level population database covering the period from 2019 to 2021, this research seeks to provide valuable insights into how COVID-19 infection may contribute to the mental health challenges faced by children and adolescents, offering a more comprehensive understanding of the pandemic's long-term impacts on youth mental health.

Materials and Methods

Data

This study was a retrospective cohort analysis utilizing data from the 2019-2021 Utah All Payers Claims Database (APCD) to investigate the causal relationship between COVID-19 infection and the incidence of depression and anxiety among school-aged children and adolescents. The APCD encompasses over 70% of Utah's population with private insurance, Medicaid, and Medicare Advantage. The APCD consists of two key files: the enrollment file and the claims file. The enrollment file includes information on insurance coverage start and end dates, birth dates,

gender, race/ethnicity, and the type of insurance (private, Medicaid, or Medicare). The claims file provides data on service/visit dates, claim types (medical, pharmacy, and dental), place of service, bill types, provider specialty, drug names, national drug codes (NDC), and healthcare costs, including reimbursed amounts and co-payments. Additionally, it includes diagnostic and procedural codes following the Current Procedural Terminology (CPT) and International Classification of Diseases, 10th Revision (ICD-10) standards. More information about the APCD can be found elsewhere [12-14]. Data used in this study were de-identified, which led to an exemption determination from the Institutional Review Board (IRB) at the University of Utah (IRB 00151091). The data were accessed between December 3, 2024, and February 15, 2025, for this research. The authors did not have access to identifiable information of the subjects during or after the data access period.

Subjects

The study population consisted of school-aged children and adolescents in Utah, aged 6 to 15 years in 2019, with continuous health insurance enrollment from 2019 through 2021. Children and adolescents who were 15 years old in 2019 were 17 years old in 2021 and remained in the school-age category.

Outcome

The primary dependent variable assessed was the occurrence of depression and anxiety in 2021, assessed as a binary variable (Yes/No). Depression and anxiety were identified using ICD-10 codes (depression: F06.31-F06.34, F32-F33.9, F34.1; anxiety: F06.4, F10.180-F10.980, F12.180-F16.980, F-18.180-F19.980, F40.00-F41.9, F93.0, F94.0) [15], in accordance with the Centers for Medicare and Medicaid Services (CMS) and Agency for Healthcare Research and Quality (AHRQ) Clinical Classifications Software Refined (CCSR) guidelines [16].

Independent variable

Two independent variables were created. The first variable was binary, indicating COVID-19 infection (Yes/No) in 2020, while the second variable had three categories reflecting the severity of COVID-19 infection: no infection, mild infection, and severe infection. COVID-19 infection was identified using a combination of ICD-10 codes (J1281, J1282, U071, U099, B948, B9729, Z8616, O985, Z20828) and CPT codes (86413, 86328, 86769, 87426, 87428, 87635, 87636, 87637, 87811, 87913, C9803) [15-17]. Severity of infection was determined by healthcare utilization in conjunction with COVID-19 diagnosis. Mild infections were classified as COVID-19 diagnoses without any emergency room (ER) visits or hospitalizations, while severe infections were defined as those associated with an ER visit or hospital admission within seven days before or after the COVID-19 diagnosis [15].

Covariates

A total of 27 chronic conditions were considered, and 15 covariates were selected using the Least Absolute Shrinkage and Selection Operator (LASSO), a machine learning approach. These covariates included age category (6-10 vs. 11-15), gender (male vs. female), race/ethnicity (non-Hispanic White, Hispanic, non-Hispanic Black, non-Hispanic Asian/Pacific Islander/American Indian, Unknown), insurance type (private insurance vs. Medicaid), sleep disorder (Yes/No), Attention-Deficit/Hyperactivity Disorder (ADHD) (Yes/No), pain diagnosis (Yes/No), blindness (Yes/No), any cancer diagnosis (Yes/No), asthma (Yes/No), chronic headaches including migraines (Yes/No), allergy reactions including anaphylaxis (Yes/No), hearing loss (Yes/No), neurocognitive disorder (Yes/No), and obesity (Yes/No).

Statistical approach

Covariate selection was conducted using LASSO, resulting in 15 variables being selected from the 27 chronic conditions considered. The selected lambda (λ) based on 10-fold cross-validation was 0.0003, with a deviance of 0.446.

All covariates, except for blindness, showed statistically significant differences between subjects with and without COVID-19 infection. To account for these differences, which could impact the incidence of depression and anxiety in 2021, Entropy Balancing (EB) was employed to equalize these baseline characteristics [15, 18]. A common approach for balancing covariates is Propensity Score Matching (PSM), which selects matched cases and controls. In contrast, EB utilizes all subjects available in the study to create weights for balancing. A study reported that EB is more effective at balancing covariates than PSM and is doubly robust [19]. Before and after EB, standardized mean differences (SMD) of the covariates were compared, with all SMD values after EB being less than 0.1, indicating good balance and effectively eliminating observed confounders that could affect the outcome (Appendix A).

Weighted summary statistics, including mean, standard deviation (SD), and percentages, were used to summarize the characteristics of the study subjects. T-tests were conducted for continuous variables, and Chi-square tests were used for categorical variables to assess statistical differences in the summary table. Weighted logistic regression was employed to identify factors associated with the incidence of depression and anxiety. The first logistic regression included subjects with and without COVID-19 infection as the independent variable, while the second logistic regression categorized subjects based on their COVID-19 symptoms: no infection, moderate symptoms, and severe symptoms. A p-value of less than 0.05 was considered statistically significant. Stata 18.5 was used for the analysis.

Results

A total of 894,884 children aged 0-17 years were identified in 2019. Among them, 352,087 had 36 months of continuous enrollment, and 217,434 children aged 6 to 15 years were selected for the study. A total of 33,159 subjects with depression or anxiety diagnoses in 2019 and 2020 were excluded. Additionally, 29,696 subjects with a COVID-19 diagnosis in 2021 were also excluded from the analysis. The final sample size for the analysis was 154,579 subjects.

After entropy weighting, the average age in 2019 was 10.8 years (SD = 2.8), with approximately 56% of subjects in the 11-15 age group. Females comprised 48% of the sample, and about 26% were covered by Medicaid. Among the subjects, 2.5% had a sleep disorder, 5% had an ADHD diagnosis, and 7.4% had an asthma diagnosis in 2019 (Table 1).

Table 1. Characteristics of subjects with and without COVID-19 infection

	COVID-19 infection			
	No	Yes	Total	p-value
N	119,432 (77.3%)	35,147 (22.7%)	154,579 (100.0%)	
Age in 2019 (continuous)	10.707 (2.805)	10.817 (2.864)	10.762 (2.835)	<0.001
Age category				0.928
6-10	52,670 (44.1%)	15,500 (44.1%)	68,169 (44.1%)	
11-15	66,762 (55.9%)	19,647 (55.9%)	86,410 (55.9%)	
Female	57,686 (48.3%)	16,976 (48.3%)	74,662 (48.3%)	0.992
Medicaid	31,291 (26.2%)	9,209 (26.2%)	40,500 (26.2%)	0.912
Race/Ethnicity				1.000
Non-Hispanic White	16,720 (14.0%)	4,921 (14.0%)	21,641 (14.0%)	
Hispanic	9,793 (8.2%)	2,882 (8.2%)	12,675 (8.2%)	
Non-Hispanic Black	717 (0.6%)	211 (0.6%)	927 (0.6%)	
Non-Hispanic others	2,030 (1.7%)	597 (1.7%)	2,628 (1.7%)	
Unknown	90,052 (75.4%)	26,501 (75.4%)	116,553 (75.4%)	
Sleep disorder	2,986 (2.5%)	879 (2.5%)	3,864 (2.5%)	0.993
ADHD	6,091 (5.1%)	1,792 (5.1%)	7,884 (5.1%)	0.994

Pain	25,320 (21.2%)	7,451 (21.2%)	32,771 (21.2%)	0.969
Blindness	2,986 (2.5%)	879 (2.5%)	3,864 (2.5%)	0.999
Cancer	8,838 (7.4%)	2,601 (7.4%)	11,439 (7.4%)	0.986
Asthma	8,838 (7.4%)	2,601 (7.4%)	11,439 (7.4%)	0.985
Headache	5,374 (4.5%)	1,582 (4.5%)	6,956 (4.5%)	0.990
Severe allergy	12,301 (10.3%)	3,620 (10.3%)	15,922 (10.3%)	0.987
Hearing loss	1,672 (1.4%)	492 (1.4%)	2,164 (1.4%)	0.993
Neurocognitive disorders	9,555 (8.0%)	2,812 (8.0%)	12,366 (8.0%)	0.994
Obesity	8,121 (6.8%)	2,390 (6.8%)	10,511 (6.8%)	0.991

Among the total subjects, 22.7% had COVID-19 infection in 2020, and 9.2% of those with COVID-19 experienced new depression or anxiety in 2021. In contrast, 5.3% of subjects without COVID-19 had new incidences of depression or anxiety in 2021. Of all subjects in 2020, 12.3% had moderate symptoms, while 10.4% had severe symptoms. Among those with moderate symptoms, 8.3% experienced new depression or anxiety, compared to 10.2% of those with severe symptoms ($p < 0.01$) (Table 2).

Table 2. Incidence of depression and anxiety in 2021 by COVID-19 infection status

	COVID-19 infection in 2020			
	No	Yes	Total	p-value
N	119,432 (77.3%)	35,147 (22.7%)	154,579 (100.0%)	
Incidence of depression or/and anxiety in 2021	6,278 (5.3%)	3,223 (9.2%)	9,501 (6.1%)	<0.001

	No COVID infection	COVID-19 infection in 2020		Total	p-value
		Moderate symptoms	Severe symptoms		
N	119,432 (77.3%)	19,066 (12.3%)	16,081 (10.40%)	154,579 (100.0%)	

Incidence of depression or/and anxiety in 2021	6,278 (5.3%)	1,587 (8.3%)	1,636 (10.20%)	9,501 (6.1%)	<0.001

After controlling for potential confounders, subjects with COVID-19 infection had a 49% higher likelihood of receiving new depression and anxiety diagnoses in 2021 (OR = 1.49, $p < 0.01$). Compared to the 6-10 age group, the 11-15 age group had 14% higher odds of new depression and anxiety (OR = 1.14, $p < 0.01$). Subjects with sleep disorders (OR = 1.43, $p < 0.01$), ADHD (OR = 1.45, $p < 0.01$), pain diagnoses (OR = 1.20, $p < 0.01$), and headaches (OR = 1.23, $p < 0.01$) had higher odds of depression and anxiety in 2021 (Table 3).

Table 3. Factors associated with the incidence of depression and anxiety

Variable	Odds ratio	p-value	95% confidence interval	
COVID-19 infection	1.49	<0.01	1.42	1.57
Age category				
6-10	reference			
11-15	1.14	<0.01	1.13	1.15
Female	1.90	<0.01	1.80	2.01
Type of insurance				
Private insurance	reference			
Medicaid	0.86	<0.01	0.80	0.93
Race/Ethnicity				
Non-Hispanic White	reference			
Hispanic	0.64	<0.01	0.57	0.71
Non-Hispanic Black	0.53	<0.01	0.38	0.76
Non-Hispanic others	0.37	<0.01	0.28	0.48
Unknown	0.70	<0.01	0.64	0.75
Sleep disorder	1.43	<0.01	1.23	1.66
ADHD	1.45	<0.01	1.22	1.73
Pain	1.20	<0.01	1.13	1.28
Blindness	1.08	0.35	0.92	1.28
Cancer	1.09	0.06	1.00	1.20
Asthma	1.14	0.01	1.03	1.26
Headache	1.23	<0.01	1.10	1.38
Severe allergy	1.14	<0.01	1.05	1.24
Hearing loss	0.99	0.90	0.78	1.24

Neurocognitive disorders	1.34	<0.01	1.15	1.57
Obesity	1.37	<0.01	1.24	1.51

Compared to those without COVID-19 infection, subjects with moderate symptoms and those with severe symptoms were 40% (OR = 1.40, $p < 0.01$) and 60% (OR = 1.60, $p < 0.01$) more likely to experience depression and anxiety, respectively. Females had a 90% higher likelihood of having depression and anxiety than males (OR = 1.90, $p < 0.01$), and those with sleep disorders had 42% higher odds of experiencing these conditions (OR = 1.42, $p < 0.01$) (Table 4).

Table 4. Association between COVID-19 infection severity and the incidence of depression and anxiety

Variable	Odds ratio	p-value	95% confidence interval	
Severity of COVID-19 infection				
No infection	reference			
Moderate infection	1.40	<0.01	1.32	1.49
Severe infection	1.60	<0.01	1.50	1.70
Age category				
6-10	reference			
11-15	1.14	<0.01	1.13	1.15
Female	1.90	<0.01	1.80	2.00
Type of insurance				
Private insurance	reference			
Medicaid	0.87	<0.01	0.80	0.94
Race/Ethnicity				
Non-Hispanic White	reference			
Hispanic	0.64	<0.01	0.57	0.72
Non-Hispanic Black	0.53	<0.01	0.38	0.75
Non-Hispanic others	0.37	<0.01	0.28	0.48
Unknown	0.71	<0.01	0.65	0.76
Sleep disorder	1.42	<0.01	1.22	1.66
ADHD	1.47	<0.01	1.23	1.75
Pain	1.19	<0.01	1.12	1.27
Blindness	1.08	0.37	0.91	1.27
Cancer	1.09	0.07	0.99	1.20
Asthma	1.14	0.01	1.03	1.26
Headache	1.23	<0.01	1.09	1.37

Severe allergy	1.14	<0.01	1.04	1.24
Hearing loss	0.97	0.81	0.77	1.23
Neurocognitive disorders	1.33	<0.01	1.14	1.55
Obesity	1.36	<0.01	1.23	1.50

Discussion

This study contributes to the small body of literature that examines mental health detriments of youth infected by COVID-19 infection. It demonstrates a relationship between COVID-19 infections among youth and elevated rates of depression and anxiety. Overall, youth with a history of COVID-19 infection exhibited 49% higher rates of mental health concerns compared to youth without infection. Children and adolescents who experienced a severe COVID-19 infection experienced 60% higher rates of depression and anxiety. There is limited information about impact of COVID-19 infection on mental illness of youth as most studies focus on the impacts of the COVID-19 infection among adult populations (≥18 years old), [20, 21]. In addition, studies have explored the broader effects of the COVID-19 pandemic on children, including such variables as school closures, reduced social interactions, changes in family dynamics, mental health, and financial instability [3, 4, 7, 22-27].

Fewer studies have examined the direct impact of COVID-19 infection on individuals, especially on children and youth. Some studies have found no significant impact of COVID-19 on children's mental health, while other studies reported that COVID-19 infection may lead to a higher likelihood of youth being diagnosed with a mental disorder. Using electronic medical records of youth from 12-17 years old in 2020 and 2021, Bilu et al. (2023) reported that infected youth had significantly lower risks in depression (hazards ratio (HR)=0.65, 95% CI: 0.53-0.80, p<0.01) and anxiety (HR=0.82, 95% CI: 0.71-0.95, p<0.01) than youth with no COVID-19 infection. Taquet et al. (2022) using a retrospective cohort study design, reported that children with and without

COVID-19 infection had no difference in incidence of anxiety disorder (HR=1.00, 95% CI: 0.94-1.06) and mood disorder (HR=1.02, 95% CI: 0.94-1.10) [28]. On the other hand, some studies have assessed the psychological impact of COVID-19 on children and adolescents, highlighting the increased risk of mental health disorders, including anxiety and depression, following infection. A study by McVoy et al. (2023) examined pediatric subjects with chronic illnesses, such as asthma and diabetes, and found that these children were more likely to develop a new psychiatric disorder following a COVID-19 infection. Specifically, the study found a positive association between asthma and the development of a new psychiatric diagnosis, with an odds ratio (OR) of 1.4 (95% CI: 1.15-1.71, $p < .001$). Children with diabetes exhibited an even stronger association, with an OR of 1.81 (95% CI: 1.11-2.94, $p = .015$) [5]. Similarly, Liu et al. (2021) explored the mental health outcomes of children who had recovered from COVID-19, finding that 31.6% of the participants reported significant anxiety symptoms, almost double the rate observed in healthy children (18.9%). Among the 38 pediatric patients they studied, 18.4% exhibited clinically significant PTSD symptoms, and 15.8% showed signs of depression. The study also highlighted a significant association between the severity of COVID-19 symptoms and the level of anxiety and depression, with children experiencing more severe symptoms tending to report higher levels of mental distress [8].

Furthermore, Solmaz et al. (2022) investigated anxiety levels in 292 pediatric COVID-19 patients (8-18 years old in 2020 and 2021) and found that 49.3% of the children reported high anxiety levels, which were primarily attributed to social isolation and the fear of death or family loss. The mean anxiety score for the participants was 25.5 (SD = 14.37), with girls (mean = 29.54) showing significantly higher anxiety levels compared to boys (mean = 21.71). The study also observed a direct correlation between the number of symptoms and anxiety scores, with children

experiencing 11 or more symptoms reporting the highest anxiety levels (mean = 30.89), compared to those with 0-5 symptoms (mean = 22.42) [29]. Strawn et al. (2023) examined differences in anxiety symptoms of adolescents (12–17-year-old in 2019) before and after COVID-19 infection. They reported the increase in anxiety symptomology to be 33% in through the years following COVID-19 infection.

Serious physical illnesses often correlate with adverse psychological outcomes [30-32]. At the onset of the pandemic, healthcare efforts primarily focused on treating the physical health symptoms from COVID-19 infection. The novelty of the virus meant that the psychological impact of the illness took a lower priority compared to the discovery of its cure, leaving a gap in mental health care. However, research has now highlighted the needs for also treating the mental health symptoms with a focus on vulnerable populations. Health crises can disproportionately impact younger populations, exacerbating mental health vulnerabilities through the stress of physical illness [33-37]. The direct experience of illness, fear of severe outcomes, the stigma associated with being infected [11, 38, 39], fatality of a loved one [40], may exacerbate the distress caused by the infection. Youth who were directed to emergency room services and hospitalizations for severe infections likely faced even more acute distress, amplifying their risk of mental health disorders [30-32]. Some individuals with COVID-19 infections may experience long periods of illness, which can persist for more than three months and pose additional challenges in managing their mental health. Additionally, fatigue, sleep disturbances, depression, anxiety, psychosis, cognitive impairment, obsessive-compulsive disorder, and post-traumatic stress disorder were some noted mental health conditions in individuals following long COVID [41]. All these contexts may act as stressors that are particularly challenging for children and adolescents, as they are in a crucial stage of development [34, 35, 42].

This study found that approximately 46% of youth with COVID-19 experienced severe infection, defined as hospital admissions or ER visits within seven days before or after infection. However, other studies, which used varying definitions of COVID-19 severity, reported lower rates. For instance, Mayo Clinic (2024) found a prevalence of 1.5% of severe cases among youth [43]. Similarly, another study reported a prevalence of 5.2%, with the majority of cases being mild or moderate [44]. In contrast, Campos et al. (2024) found a prevalence of 28%, with significant variations in disease severity based on comorbidities and other factors [45]. Unlike these prior studies, the findings suggest that the proportion of youth with severe COVID-19 infection may be higher than previously reported. This discrepancy highlights the potential for an underestimation of mental illness among children and adolescents affected by the pandemic. For example, in Campos et al. (2024), the presence of dyspnea was significantly associated with more severe disease, with an odds ratio (OR) of 6.3 (95% CI: 2.8-14.3), while fever (OR=3.8; 95% CI: 2.0-7.4) and cough (OR=3.4; 95% CI: 2.0-6.0) were similarly linked to greater disease severity [45]. These findings underscore the need for further investigation into the mental health impacts on children and adolescents, particularly those with severe COVID-19 infection or comorbid conditions. Such insights are crucial for developing comprehensive care strategies for this vulnerable population.

The purpose of examining a population-level data such as Utah APCD was to highlight more nuanced impacts to children's mental health from COVID-19 infection and characterize the need for mental health support and a broader public health response to care for vulnerable populations [46]. This study exhibits the bio-psycho-social impact of COVID-19 and should serve to inform the continued treatment of COVID-19 as a holistic process. Healthcare providers, educators, and policymakers must recognize that the psychological impact of COVID-19 infections extends beyond the acute phase of infection, particularly for those who experienced severe illness.

Increased funding for mental health services is essential to meet the growing demand for support. Mental health interventions tailored for youth, especially those recovering from severe COVID-19 infections, are essential to mitigate the long-term psychological consequences. Early identification of mental health needs, particularly in children with severe infections, is essential to prevent long-term impairments and promote recovery. Sufficient and equitable care should be allotted to all parties suffering from the COVID-19 pandemic.

Additional consequences brought on by COVID-19 infection, such as missing school days, reduced social interactions, and the challenge of catching up academically upon returning to school, likely compound the psychological impact of COVID-19 infection [3, 4, 22, 47, 48]. Schools and community organizations must play a pivotal role in supporting students with mental health challenges considering this current body of research [43, 49, 50]. For instance, developing inclusive programs that offer psychological counseling, peer support, academic interventions, and mental health education may help foster resilience and academic success in youth affected by the COVID-19 infection. Additionally, those providing mental health interventions should account for the unique aspects of the population they serve. Factors such as the severity of COVID-19 infection, the age of youths, and the disproportionate impact of mental illness on racial and ethnic minorities must be considered [51-55].

This research fills a specific gap within the literature which is invaluable to informing medical providers and school personnel. However, several limitations must be acknowledged. First, the study relies on claims data, which may not capture subclinical symptoms of depression and anxiety that do not result in formal diagnoses. Second, the reliance on ICD-10 codes for identifying mental health conditions could introduce some degree of misclassification. Another important limitation is that medications were not used to identify or verify comorbid conditions,

which may have resulted in underreporting or incomplete identification of health conditions including mental disorders. In addition, many children and adolescents may experience no symptoms despite having COVID-19 infections, resulting in the absence of COVID-19-related diagnosis and procedure codes. Consequently, there may be an underestimation of the number of individuals with COVID-19 infections. The generalizability of the findings may also be limited to Utah's population, as the data primarily reflects healthcare access and policies specific to the state. Further research across different regions would be necessary to confirm whether similar associations exist in other states or populations with different demographic or healthcare characteristics. Furthermore, there were considerable amounts of missing data on race and ethnicity, which limits the ability to explore mental health outcomes across these important demographic factors. While efforts were made to account for various confounders through covariates, the study cannot entirely rule out the influence of unmeasured factors such as family history of mental illness, parental stress, or community-level stressors. Lastly, as the data only extends through 2021, the long-term psychological impact of COVID-19 on children and adolescents remains uncertain.

Conclusions

This study provides evidence that COVID-19 infection, particularly severe cases, is associated with a higher incidence of depression and anxiety among children and adolescents. These findings highlight the need for targeted mental health interventions for youth affected by the pandemic. As society continues to navigate the post-pandemic landscape, prioritizing the mental well-being of younger populations is critical for fostering resilience and ensuring that adequate resources are available to support their psychological recovery. Addressing these

challenges now will not only benefit individual children and adolescents but will also contribute to the broader goal of promoting public mental health in future generations.

Financial disclosure

Support for all datasets in the study was provided by the University of Utah Matheson Center for Health Care Studies.

Ethics statement

The study was conducted in accordance with the Declaration of Helsinki, and approved by the Institutional Review Board (or Ethics Committee) of University of Utah (protocol code IRB_00151091 and November 09, 2023).

Informed Consent Statement

Patient consent was waived for this study because it poses minimal risk to participants and aligns with ethical guidelines for research.

Competing interests

The authors declare no conflicts of interest.

Data Availability

Data cannot be shared publicly because of the regulations of Utah Department of Health and Human Services, and the Utah Resource for Genetic and Epidemiologic Research (RGE). Data are available from the University of Utah Institutional Data Access / Ethics Committee (contact via RGE) for researchers who meet the criteria for access to confidential data.

References

1. Mental Health America. The State of Mental Health in America 2019. Mental Health America; 2019. [Available from: mhanational.org/issues/2019/mental-health-america-youth-data.

2. Mental Health America. The State of Mental Health in America 2023. Mental Health America; 2023. [Available from: mhanational.org/issues/2023/mental-health-america-youth-data.

3. Larsen L, Helland MS, Holt T. The impact of school closure and social isolation on children in vulnerable families during COVID-19: a focus on children's reactions. Eur Child Adolesc Psychiatry. 2022;31(8):1-11.

4. Loades ME, Chatburn E, Higson-Sweeney N, Reynolds S, Shafran R, Brigden A, et al. Rapid Systematic Review: The Impact of Social Isolation and Loneliness on the Mental Health of Children and Adolescents in the Context of COVID-19. J Am Acad Child Adolesc Psychiatry. 2020;59(11):1218-39.e3.

5. McVoy M, Hung R, Bransteter I, Perez JA, Segall TL, Surdam JO, et al. Impact of SARS-CoV-2 (COVID-19) Infections on Mental Health Diagnoses in Youth With Chronic Illness. JAACAP Open. 2023;2(3):180-7.

6. Zhang-James Y, Clay JWS, Aber RB, Gamble HM, Faraone SV. Post–COVID-19 Mental Health Distress in 13 Million Youth: A Retrospective Cohort Study of Electronic Health Records. Journal of the American Academy of Child & Adolescent Psychiatry. 2024.

7. Nearchou F, Flinn C, Niland R, Subramaniam SS, Hennessy E. Exploring the Impact of COVID-19 on Mental Health Outcomes in Children and Adolescents: A Systematic Review. Int J Environ Res Public Health. 2020;17(22):8479.

8. Liu D, Liu W, Rodriguez M, Zhang J, Zhang F. The Mental Health Impacts of COVID-19 on Pediatric Patients Following Recovery. Front Psychol. 2021;12:628707-.

9. Brasso C, Bellino S, Blua C, Bozzatello P, Rocca P. The Impact of SARS-CoV-2 Infection on Youth Mental Health: A Narrative Review. Biomedicines. 2022;10(4):772.

10. Bilu Y, Flaks-Manov N, Goldshtein I, Bivas-Benita M, Akiva P, Bodenheimer G, et al. Youth Mental Health Outcomes up to Two Years After SARS-CoV-2 Infection Long-COVID or Long-Pandemic Syndrome: A Retrospective Cohort Study. Journal of Adolescent Health. 2023;73(4):701-6.

11. Samji H, Wu J, Ladak A, Vossen C, Stewart E, Dove N, et al. Review: Mental health impacts of the COVID-19 pandemic on children and youth - a systematic review. Child Adolesc Ment Health. 2022;27(2):173-89.

12. Kim J, Simper S, McKinlay R, Cottam D, Surve A, Adams T. Healthcare cost and utilization of bariatric surgical patients with and without preoperative mental health diagnoses. Surg Obes Relat Dis. 2020;16(5):682-9.

13. Kim J, Waitzman N, Richards N, Adams T. Prescriptions for pain medication before and after bariatric surgery. Surg Endosc. 2022;36(7):4960-8.

14. Kim J, Waitzman N, Simper S, McKinlay R, Cottam D, Surve A, et al. Effects of Post-operative Nutritional Disorders Following Bariatric Surgery on Health Care Cost and Use. Obes Surg. 2021;31(6):2503-10.

15. Kim J, Ben-Umeh KC, Weir R, Manotas K, Kleinschmit K, Fischer A, et al. Evaluating the risk of sleep disorders in subjects with a prior COVID-19 infection. PLoS One. 2024;19(10):e0311929.

16. Clinical Classifications Software Refined (CCSR) for ICD-10-CM Diagnoses: Agency for Healthcare Research and Quality.; [Available from: https://hcup-us.ahrq.gov/toolssoftware/ccsr/dxccsr.jsp.

17. Illinois Medicaid COVID-19 Fee Schedule: llinois Department of Healthcare and Family Services; 2024 [Available from: https://hfs.illinois.gov/content/dam/soi/en/web/hfs/sitecollectiondocuments/COVID19FeeScheduleEffective03132023.pdf.

18. Hainmueller J. Entropy balancing for causal effects: A multivariate reweighting method to produce balanced samples in observational studies. Political analysis. 2012;20(1):25-46.

19. Zhao Q, Percival D. Entropy balancing is doubly robust. De Gruyter; 2017. p. 20160010.

20. Penninx B, Benros ME, Klein RS, Vinkers CH. How COVID-19 shaped mental health: from infection to pandemic effects. Nat Med. 2022;28(10):2027-37.

21. Xie Y, Xu E, Al-Aly Z. Risks of mental health outcomes in people with covid-19: cohort study. BMJ. 2022;376:e068993-e.

22. Cohodes EM, McCauley S, Gee DG. Parental Buffering of Stress in the Time of COVID-19: Family-Level Factors May Moderate the Association Between Pandemic-Related Stress and Youth Symptomatology. Res Child Adolesc Psychopathol. 2021;49(7):935-48.

23. Bierman A, Upenieks L, Glavin P, Schieman S. Accumulation of economic hardship and health during the COVID-19 pandemic: Social causation or selection? Soc Sci Med. 2021;275:113774-.

24. Calvano C, Engelke L, Di Bella J, Kindermann J, Renneberg B, Winter SM. Families in the COVID-19 pandemic: parental stress, parent mental health and the occurrence of adverse childhood experiences-results of a representative survey in Germany. Eur Child Adolesc Psychiatry. 2022;31(7):1-13.

25. Cielo F, Ulberg R, Di Giacomo D. Psychological Impact of the COVID-19 Outbreak on Mental Health Outcomes among Youth: A Rapid Narrative Review. Int J Environ Res Public Health. 2021;18(11):6067.

26. Kauhanen L, Wan Mohd Yunus WMA, Lempinen L, Peltonen K, Gyllenberg D, Mishina K, et al. A systematic review of the mental health changes of children and young people before and during the COVID-19 pandemic. Eur Child Adolesc Psychiatry. 2023;32(6):995-1013.

27. Oliveira JMDd, Butini L, Pauletto P, Lehmkuhl KM, Stefani CM, Bolan M, et al. Mental health effects prevalence in children and adolescents during the COVID-19 pandemic: A systematic review. Worldviews Evid Based Nurs. 2022;19(2):130-7.

28. Taquet M, Sillett R, Zhu L, Mendel J, Camplisson I, Dercon Q, et al. Neurological and psychiatric risk trajectories after SARS-CoV-2 infection: an analysis of 2-year retrospective cohort studies including 1 284 437 patients. Lancet Psychiatry. 2022;9(10):815-27.

29. Solmaz A, Karataş H, Ercan TMF, Erat T, Solmaz F, Kandemir H. Anxiety in Paediatric Patients Diagnosed with COVID-19 and the Affecting Factors. J Trop Pediatr. 2022;68(2):fmac018.

30. Delamater AM, Guzman A, Aparicio K. Mental health issues in children and adolescents with chronic illness. International Journal of Human Rights in Healthcare. 2017;10(3):163-73.

31. Stockbridge EL, Wilson FA, Pagán JA. Psychological Distress and Emergency Department Utilization in the United States: Evidence from the Medical Expenditure Panel Survey. Academic Emergency Medicine. 2014;21(5):510-9.

32. Yıldırım N, Dayılar Candan H, İnan AH. Psychological Distress in Patients with COVID-19 During Hospitalization. Clinical Nursing Research. 2021;31(3):376-84.

33. Tso WWY, Wong RS, Tung KTS, Rao N, Fu KW, Yam JCS, et al. Vulnerability and resilience in children during the COVID-19 pandemic. Eur Child Adolesc Psychiatry. 2022;31(1):161-76.

34. Schmaal L, Yücel M, Ellis R, Vijayakumar N, Simmons JG, Allen NB, et al. Brain Structural Signatures of Adolescent Depressive Symptom Trajectories: A Longitudinal Magnetic Resonance Imaging Study. Journal of the American Academy of Child & Adolescent Psychiatry. 2017;56(7):593-601.e9.

35. Bos MGN, Peters S, van de Kamp FC, Crone EA, Tamnes CK. Emerging depression in adolescence coincides with accelerated frontal cortical thinning. J Child Psychol Psychiatry. 2018;59(9):994-1002.

36. Luby JL, Belden AC, Jackson JJ, Lessov-Schlaggar CN, Harms MP, Tillman R, et al. Early Childhood Depression and Alterations in the Trajectory of Gray Matter Maturation in Middle Childhood and Early Adolescence. JAMA Psychiatry. 2016;73(1):31-8.

37. Stevanovic D, Kabukcu Basay B, Basay O, Leskauskas D, Nussbaum L, Zirakashvili M. COVID-19 pandemic-related aspects and predictors of emotional and behavioural symptoms in youth with pre-existing mental health conditions: results from Georgia, Lithuania, Romania, Serbia, and Turkey. Nordic Journal of Psychiatry. 2021;76(7):515-22.

38. Hinshaw SP. The stigmatization of mental illness in children and parents: developmental issues, family concerns, and research needs. Journal of Child Psychology and Psychiatry. 2005;46(7):714-34.

39. Adegboye D, Williams F, Collishaw S, Shelton K, Langley K, Hobson C, et al. Understanding why the COVID-19 pandemic-related lockdown increases mental health difficulties in vulnerable young children. JCPP Adv. 2021;1(1):e12005-e.

40. Koçak O, Koçak ÖE, Younis MZ. The Psychological Consequences of COVID-19 Fear and the Moderator Effects of Individuals' Underlying Illness and Witnessing Infected Friends and Family. Int J Environ Res Public Health. 2021;18(4):1836.

41. HHS Issues Advisory on Mental Health Symptoms and Conditions Related to Long COVID: U.S. Department of Health and Human Services; 2023 [Available from: https://www.hhs.gov/about/news/2023/06/21/hhs-issues-advisory-mental-health-symptoms-and-conditions-related-long-covid.html.

42. Masarik AS, Conger RD. Stress and child development: a review of the Family Stress Model. Current Opinion in Psychology. 2017;13:85-90.

43. COVID-19 in babies and children: Mayo Clinic; 2024 [Available from: https://www.mayoclinic.org/diseases-conditions/coronavirus/in-depth/coronavirus-in-babies-and-children/art-20484405

44. Ludvigsson JF. Systematic review of COVID-19 in children shows milder cases and a better prognosis than adults. Acta Paediatr. 2020;109(6):1088-95.

45. Campos CG, Muniz LA, Belo VS, Costa CC, Borges Neto JdS, Novais RLR, et al. Characteristics and clinical outcomes of adolescents infected by SARS-CoV-2: a systematic review. Revista Paulista de Pediatria. 2024;42.

46. Palinkas LA, De Leon J, Salinas E, Chu S, Hunter K, Marshall TM, et al. Impact of the COVID-19 Pandemic on Child and Adolescent Mental Health Policy and Practice Implementation. Int J Environ Res Public Health. 2021;18(18):9622.

47. Engzell P, Frey A, Verhagen MD. Learning loss due to school closures during the COVID-19 pandemic. Proc Natl Acad Sci U S A. 2021;118(17):e2022376118.

48. Harmey S, Moss G. Learning disruption or learning loss: using evidence from unplanned closures to inform returning to school after COVID-19. Educational Review. 2021;75(4):637-56.

49. Gómez G, Basagoitia A, Burrone MS, Rivas M, Solís-Soto MT, Dy Juanco S, et al. Child-Focused Mental Health Interventions for Disasters Recovery: A Rapid Review of Experiences to Inform Return-to-School Strategies After COVID-19. Front Psychiatry. 2021;12:713407-.

50. Mariani Wigley ILC, Mascheroni E, Bulletti F, Bonichini S. COPEWithME: The Role of Parental Ability to Support and Promote Child Resilient Behaviors During the COVID-19 Emergency. Front Psychol. 2021;12:732745-.

51. Mpofu JJ, Cooper AC, Ashley C, Geda S, Harding RL, Johns MM, et al. Perceived Racism and Demographic, Mental Health, and Behavioral Characteristics Among High School Students During the COVID-19 Pandemic - Adolescent Behaviors and Experiences Survey, United States, January-June 2021. MMWR Suppl. 2022;71(3):22-7.

52. Penner F, Rajesh A, Kinney KL, Mabus KL, Barajas KG, McKenna KR, et al. Racial and demographic disparities in emergency department utilization for mental health concerns before and during the COVID-19 pandemic. Psychiatry Res. 2022;310:114442-.

53. Rega A, Nappo R, Simeoli R, Cerasuolo M. Age-Related Differences in Psychological Distress during the COVID-19 Pandemic. Int J Environ Res Public Health. 2022;19(9):5532.

54. Schmidt SJ, Barblan LP, Lory I, Landolt MA. Age-related effects of the COVID-19 pandemic on mental health of children and adolescents. Eur J Psychotraumatol. 2021;12(1):1901407-.

55. Shi L, Zhang D, Martin E, Chen Z, Li H, Han X, et al. Racial Discrimination, Mental Health and Behavioral Health During the COVID-19 Pandemic: a National Survey in the United States. J Gen Intern Med. 2022;37(10):2496-504.

Appendix A. Standardized mean differences before and after entropy balancing

Before entropy balancing

	Subjects with COVID-19 infection			Subjects with no COVID-19 infection			
	mean	variance	skewness	mean	variance	skewness	standardized mean difference
Age category	0.559	0.247	-0.239	0.466	0.249	0.138	0.189
Female	0.483	0.250	0.069	0.473	0.249	0.108	0.019
Hispanic	0.082	0.075	3.048	0.097	0.088	2.726	-0.054
Non-Hispanic Black	0.006	0.006	12.720	0.011	0.011	9.349	-0.064
Non-Hispanic others	0.017	0.017	7.374	0.018	0.018	7.269	-0.004
Unknown	0.755	0.185	-1.183	0.729	0.198	-1.029	0.060
Medicaid	0.262	0.193	1.083	0.330	0.221	0.722	-0.155
Sleep disorder	0.025	0.024	6.106	0.017	0.017	7.429	0.049
ADHD	0.051	0.048	4.103	0.044	0.042	4.446	0.030
Pain	0.212	0.167	1.409	0.148	0.126	1.985	0.157
Blindness	0.025	0.024	6.094	0.024	0.024	6.161	0.003
Cancer	0.074	0.069	3.253	0.057	0.054	3.822	0.065
Asthma	0.074	0.069	3.249	0.046	0.044	4.329	0.107
Headache	0.045	0.043	4.404	0.031	0.030	5.415	0.067
Severe allergy	0.103	0.092	2.610	0.080	0.073	3.109	0.078
Hearing loss	0.014	0.014	8.223	0.009	0.009	10.140	0.040
Neurocognitive disorders	0.080	0.074	3.086	0.072	0.067	3.321	0.032
Obesity	0.068	0.063	3.445	0.055	0.052	3.899	0.050

Appendix A. Standardized mean differences before and after entropy balancing (continued)

After entropy balancing

	Subjects with COVID-19 infection			Subjects with no COVID-19 infection			
	mean	variance	skewness	mean	variance	skewness	standardized mean difference
Age category	0.559	0.247	-0.239	0.559	0.247	-0.238	0.001
Female	0.483	0.250	0.069	0.483	0.250	0.069	0.000
Hispanic	0.082	0.075	3.048	0.082	0.075	3.047	0.000
Non-Hispanic Black	0.006	0.006	12.720	0.006	0.006	12.670	-0.001
Non-Hispanic others	0.017	0.017	7.374	0.017	0.017	7.373	0.000
Unknown	0.755	0.185	-1.183	0.754	0.185	-1.182	0.000
Medicaid	0.262	0.193	1.083	0.262	0.194	1.081	-0.001
Sleep disorder	0.025	0.024	6.106	0.025	0.024	6.108	0.000
ADHD	0.051	0.048	4.103	0.051	0.048	4.103	0.000
Pain	0.212	0.167	1.409	0.212	0.167	1.409	0.000
Blindness	0.025	0.024	6.094	0.025	0.024	6.094	0.000
Cancer	0.074	0.069	3.253	0.074	0.069	3.254	0.000
Asthma	0.074	0.069	3.249	0.074	0.069	3.250	0.000
Headache	0.045	0.043	4.404	0.045	0.043	4.405	0.000
Severe allergy	0.103	0.092	2.610	0.103	0.092	2.611	0.000
Hearing loss	0.014	0.014	8.223	0.014	0.014	8.225	0.000
Neurocognitive disorders	0.080	0.074	3.086	0.080	0.074	3.086	0.000
Obesity	0.068	0.063	3.445	0.068	0.063	3.446	0.000
